# Exploring factors for antibiotic over-prescription in children with acute upper respiratory tract infections in Assiut, Egypt: a qualitative study

**DOI:** 10.1186/s13756-023-01357-2

**Published:** 2024-01-07

**Authors:** Mahmoud Attia AbdEl-Aty, Mariam Taher Amin, Sabra Mohamed Ahmed, Ghada Omar Elsedfy, Amira Fathy El-Gazzar

**Affiliations:** 1 Department of Public Health and Community Medicine, Faculty of Medicine, Assiut University, Assiut, 71515 Egypt; 2 Department of Pediatrics, Faculty of Medicine, Children’s Hospital, Assiut University, Assiut, Egypt; 3 Department of Public Health and Community Medicine, Badr University in Cairo, Badr City, Egypt

**Keywords:** Antibiotic over-prescription, Antibiotic resistance, Antibiotic prescribing behavior

## Abstract

**Background:**

Over-prescription of antibiotics contributes to antibiotic resistance, which is a global health threat. Egypt has alarmingly high rates of antibiotic over-prescription for acute upper respiratory tract infections (URIs) in children. To effectively address this issue, it is important to understand the various factors that influence prescription behaviors. The Teixeira antibiotic prescription behavioral model (TAPBM) offers a comprehensive framework through which these factors can be explored. This qualitative study sought to investigate the perspectives of key stakeholders involved in pediatric healthcare in Egypt, with the primary goal of identifying the underlying determinants that contributed to this problem.

**Methods:**

This qualitative study was conducted in Assiut City, Egypt, between January and March 2023. Purposive sampling was used to select participants, including consultant pediatricians, supervisors of pediatric training programs, and specialists in infection prevention and control. Thirteen semi-structured in-depth interviews (IDIs) were conducted, audio-recorded, and transcribed. Thematic analysis was performed using MAXQDA 2020 software.

**Results:**

Two main themes emerged from the analysis: intrinsic factors related to physicians, extrinsic factors related to patients, and nonphysician factors. Intrinsic factors encompass personal characteristics and attitudes. Prescribing decisions were influenced by factors such as fear of complications, limited follow-up visits, and competition. Knowledge and education also played a significant role. Moreover, diagnostic uncertainty in distinguishing between bacterial and viral infections posed a challenge. Extrinsic factors included patient and caregiver factors, such as parental expectations and demands for antibiotics, driven by the belief that they produced rapid results. Moreover, patients’ demographic factors, including socioeconomic status and living conditions, affected their prescribing behavior. Health system-related factors, such as the type of healthcare institution and the absence of formal national guidelines, were identified as influential factors. Additionally, this study highlighted the influence of the pharmaceutical industry. The potential impact of the COVID-19 pandemic on antibiotic prescriptions was addressed.

**Conclusions:**

The study highlights the intricate interplay between intrinsic and extrinsic factors that shape antibiotic prescription decisions, underscoring the significance of addressing these factors in mitigating overprescribing.

**Supplementary Information:**

The online version contains supplementary material available at 10.1186/s13756-023-01357-2.

## Background

Antibiotic over-prescription is a critical issue contributing to the escalating threat of antibiotic resistance and presents substantial challenges to global health security. Both developed, and developing countries have witnessed an annual increase in the prevalence of resistant microbial strains that affect human and animal populations [1, 2]. In light of this growing problem, policymakers, health organizations, and research institutes have emphasized the necessity of implementing stricter control measures regarding antibiotic distribution and utilization within society, with a particular focus on frontline prescribers and dispensers [3, 4].

Pediatric care worldwide often includes antibiotic administration, which is crucial for treating bacterial infections in children. However, excessive prescription of antibiotics to children, particularly in cases of viral infections, is a significant public health concern and a major contributor to antibiotic resistance [5, 6]. This poses a serious threat to global progress in child health. Low- and middle-income countries (LMICs) are especially susceptible to antimicrobial resistance (AMR) due to weak pharmaceutical governance, a higher prevalence of infectious diseases, and lower standards of sanitation and public health [7, 8].

Studies conducted in Egypt have revealed widespread use of antibiotics in children with acute upper respiratory tract infections. In a study in the Minya district, pharmacists and physicians from governmental and private hospitals were surveyed regarding antibiotic prescription practices. The results showed that 64% of physicians and 81% of pharmacists frequently or occasionally prescribed antibiotics (for 3–5 days) in common cold cases [9]. Another study conducted in the Assiut District of Upper Egypt examined antibiotic prescriptions by non-specialized physicians for managing acute URIs in children. Of the 612 vignettes assessed, 326 included antibiotic prescriptions, accounting for 53.3% of cases. Antibiotic prescriptions were appropriate in only 8.3% of cases [10].

While these studies provide insights into antibiotic prescription practices in the Upper Egypt governorates of Minya and Assiut, it is important to note that antibiotic use in children with acute URIs is a matter of concern across various regions in Egypt. The limited availability of comprehensive data from other governorates, necessitates further research to gain a more holistic understanding of this issue nationwide.

Antibiotic over-prescription is a crucial public health concern that contributes to the escalation of antibiotic resistance and poses a global threat to health. Despite efforts to promote rational drug use, inappropriate and excessive antibiotic use has increased [11]. Therefore, it is essential to investigate the various factors that influence prescription behavior. Antibiotic prescription decisions are often complicated and influenced by multiple factors [12]. Therefore, to change prescribing behavior, it is crucial to gain a comprehensive understanding of antibiotic prescribing practices and the factors influencing this behavior. One of the main theories that helps comprehend physicians’ antibiotic prescription behavior is the Teixeira antibiotic prescription behavioral model (TAPBM). This model builds upon the widely accepted knowledge-attitude-practices (KAP) theory and incorporates considerations of external determinants that impact antibiotic prescription practices [13]. Antibiotic prescribing practices are influenced by a range of factors, including intrinsic factors related to the prescriber as well as external factors such as patients and the institutional environment [12].

To date, substantial knowledge gaps persist in Egypt, particularly in qualitative studies that could enhance our understanding of physicians’ prescribing behaviors. These gaps present challenges to the implementation of evidence-based interventions aimed at addressing prescriber behavior. Building on the findings of our previous study conducted in Assiut District, which highlighted a high rate of inappropriate antibiotic prescriptions (91.7%) for childhood upper respiratory infections [10], there is a pressing need for further investigation into the factors that contribute to antibiotic over-prescription in this context.

This qualitative study aimed to delve into these factors from the perspective of diverse healthcare providers engaged in pediatric healthcare within Assiut District, one of the largest districts in Upper Egypt. By employing TAPBM as a theoretical framework, this study sought to advance our understanding of the underlying factors and offer valuable insights into targeted interventions that promote the rational utilization of antibiotics. Furthermore, the findings of this research may offer insights to healthcare providers and policymakers in LMICs that share a similar context to Egypt, enabling them to comprehend this issue and formulate studies and interventions aimed at enhancing antibiotic prescription practices.

## Methods

### Study design

A qualitative study was conducted to explore the perspectives of healthcare providers, specifically physicians who engaged in supervision and mentoring of pediatrician practices regarding factors contributing to antibiotic over-prescription. The study adhered to the quality standards outlined in the Consolidated Criteria for Reporting Qualitative Research (COREQ) Guide [14] (Additional file 1).

### Interview and data collection procedure

This research was conducted from January to March 2023 in Assiut City, employing purposive sampling as the chosen sampling strategy. The purpose of this sampling approach was to deliberately capture a diverse array of perspectives. Specifically, we sought to engage individuals who held significant roles in the supervision and training of physicians dealing with pediatric cases, including consultants, experts, and individuals with policymaking responsibilities.

Regarding the participant recruitment process, we approached potential participants by initially listing individuals in these roles and then contacting them either by phone or in person at their workplace to introduce the study’s objectives. After gaining their agreement to participate, we scheduled interviews at a convenient time and place.

It is important to note that our inclusion criteria were deliberately designed to be non-restrictive, with no limitations pertaining to age, gender, or years of professional practice. Instead, the primary criterion for inclusion focused on the participants’ pivotal roles within the domain of physician supervision and training, thereby ensuring a comprehensive representation of this specific stakeholder group.

Semi-structured IDIs were conducted by MA to gather the data for this study. Data saturation, indicating that no new information or themes were identified, was achieved after nine consecutive interviews, and this saturation was subsequently confirmed by the remaining participants.

A semi-structured interview guide was used to conduct interviews (Additional file 2). This guide was developed based on a comprehensive literature review of factors influencing antibiotic over-prescription and TAPBM [15].

The interviews were conducted face-to-face by the primary researcher at the participants’ workplaces in Arabic. The interviews lasted for 20–40 min. Written consent was obtained from all participants, and the interviews were audio-recorded for accurate transcription and analysis.

### Data analysis

All IDIs were transcribed verbatim and anonymized to ensure confidentiality. The interviewer reviewed the transcripts against the audio recordings to verify their completeness and accuracy.

For data analysis, the researchers employed the MAXQDA 2020 qualitative data analysis software. The analysis was initially conducted in Arabic, and subsequently, themes and sub-themes, along with relevant quotes, were translated into English. Two researchers (M.T.A. and A.F.E.) collaborated in the analysis. Thematic analysis was employed to identify recurring themes, which were then categorized into preselected theoretically driven domains to aid in identifying factors influencing antibiotic prescribing behavior. The researchers followed the guidelines outlined by Braun and Clarke for thematic analysis [16].

The researchers followed a systematic approach to data analysis, involving multiple steps. First, they familiarized themselves with the interview material by reading and reviewing the transcripts multiple times (Step 1: Familiarization). Second, they selected significant quotes from the transcripts and assigned the appropriate codes. This process resulted in 155 codes (Step 2: Code generation).

Next, deductive thematic analysis was conducted, combining similar codes to create major categories using a thematic map based on TAPBM [15]. This process led to identifying themes and sub-themes (Step 3: Constructing themes).

The researchers reviewed and checked the themes against the entire dataset, providing appropriate names for each theme and subtheme that accurately reflected their meaning (Steps 4 and 5: Revising and defining the themes).

Finally, the results were documented, and the themes were continuously evaluated and refined if necessary to ensure that they effectively addressed the research question. The quotes used in the report were anonymized and presented to illustrate the core meaning of the themes (Step 6: Producing the report).

### Rigor criteria

This study followed the quality criteria of Lincoln and Guba (1985) to enhance the rigor and validity of the findings.

Credibility was established through member checking, inter-coder agreement, and peer debriefing. Member checking involved presenting the findings to five participants who were randomly selected from the list of participants to verify that the interpretations were consistent with their experiences and perspectives. Inter-coder agreement involved comparing the researchers’ coding of the transcripts and resolving discrepancies through discussion. Peer debriefing involved sharing the findings and interpretations with colleagues to receive feedback and to ensure that the interpretations were consistent with the data.

Transferability was enhanced by providing a detailed description of the study context, methods, and participants. The study included a diverse group of healthcare providers, which may increase the transferability of the findings to similar populations.

Reflexivity was inherent in this process through the awareness and consideration of the interviewers’ professional backgrounds. As a physician with an interest in the topic of antimicrobial resistance, her prior medical practice led her to assume that there is an over-prescription of antibiotics, primarily due to physicians’ prescribing behavior. The awareness of this preconception made the research team more focused during interviews and analyses to avoid bias.

### Ethical considerations

The study protocol was approved by the Research Review Board and Ethical Review Board of the Faculty of Medicine, Assiut University (IRB number 17,200,370). This study was registered at clinicaltrials.gov (identifier NCT04127682). During the interviews, strict adherence to ethical guidelines and regulations was ensured. The participants were fully informed about the study objectives and interview procedures, and informed consent was obtained prior to participation. To safeguard participant privacy, no personal information was collected from audio recordings or coded data. All data were stored in password-protected files, and the interview transcripts were assigned anonymous identifiers during the coding process to maintain participant confidentiality.

## Results

### Characteristics of participants

The study sample comprised physicians who engaged in supervision and mentoring of pediatrician practices. This included consultant pediatricians who held supervisory roles or collaborated with physicians in outpatient clinics at major hospitals in Assiut, including the Ministry of Health and university hospitals. Furthermore, the sample consisted of heads of pediatric departments in assistant hospitals, supervisors of training programs for pediatricians (master’s degree and Egyptian Pediatric Fellowship), infection prevention and control specialists (IPC) working in hospitals, and the health directorate.

For more details regarding participants’ characteristics, please refer to Table 1.

**Table 1:** Characteristics of participants

ID	Age	Sex	Years of experience	Position	Type of institute	Postgraduate Studies	Policymaker Role
**P01**	37	Female	11	Consultant	General Hospital	Pediatrics Egyptian Fellowship	Supervisor of a pediatrics outpatient clinic
**P02**	40	Female	15	Consultant	General Hospital	Master’s in Pediatrics	Supervision and monitoring of pediatric residents’ work
**P03**	47	Male	17	Consultant	University Teaching Hospital	MD in Pediatrics	Hospital quality assurance unit
**P04**	46	Male	19	Consultant	General Hospital	Egyptian Fellowship in Pediatrics	Trainer on Egyptian Pediatric Fellowship
**P05**	35	Male	10	Specialist	General Hospital	Master’s degree in Pediatrics	Supervisor of an outpatient clinic
**P06**	36	Male	9	Specialist	General Hospital	Egyptian Fellowship in Pediatrics	Supervisor of an emergency unit
**P07**	52	Male	24	Consultant	General Hospital	Master’s degree in Pediatrics	Head of a pediatrics department
**P08**	44	Female	18	Consultant	University teaching hospital	MD degree in Clinical Pathology (Microbiology)	Infection control unit
**P09**	46	Female	13	Infection control specialist	General Hospital	Master’s degree in Microbiology	Infection control specialist
**P10**	50	Male	24	Infection control specialist	Directorate of health	Bachelor of Pharmacy, IPC Professional Diploma	Supervisor of infection prevention and control
**P11**	65	Female	37	Consultant	University Teaching Hospital	MD degree in Pediatrics	Consultant in a teaching hospital
**P12**	58	Female	31	Consultant	University Teaching Hospital	MD degree in Pediatrics	PICU
**P13**	55	Male	28	Consultant	University Teaching Hospital	MD degree in Pediatrics	Director of a hospital

Initially, all participants acknowledged the widespread problem of antibiotics over-prescription. They recognized that this practice contributes to a rise in antibiotic resistance, which hinders treatment effectiveness and exacerbates the challenge of managing serious bacterial infections. Moreover, the participants expressed concerns about the adverse effects of antibiotics on their health.

One participant stated, *“Today, we are facing a very significant problem: children who are admitted to intensive care units and inpatients are developing infections that we have never seen before, and these infections have become resistant to the medications we typically prescribe”* (P05).

**Fig. 1:**
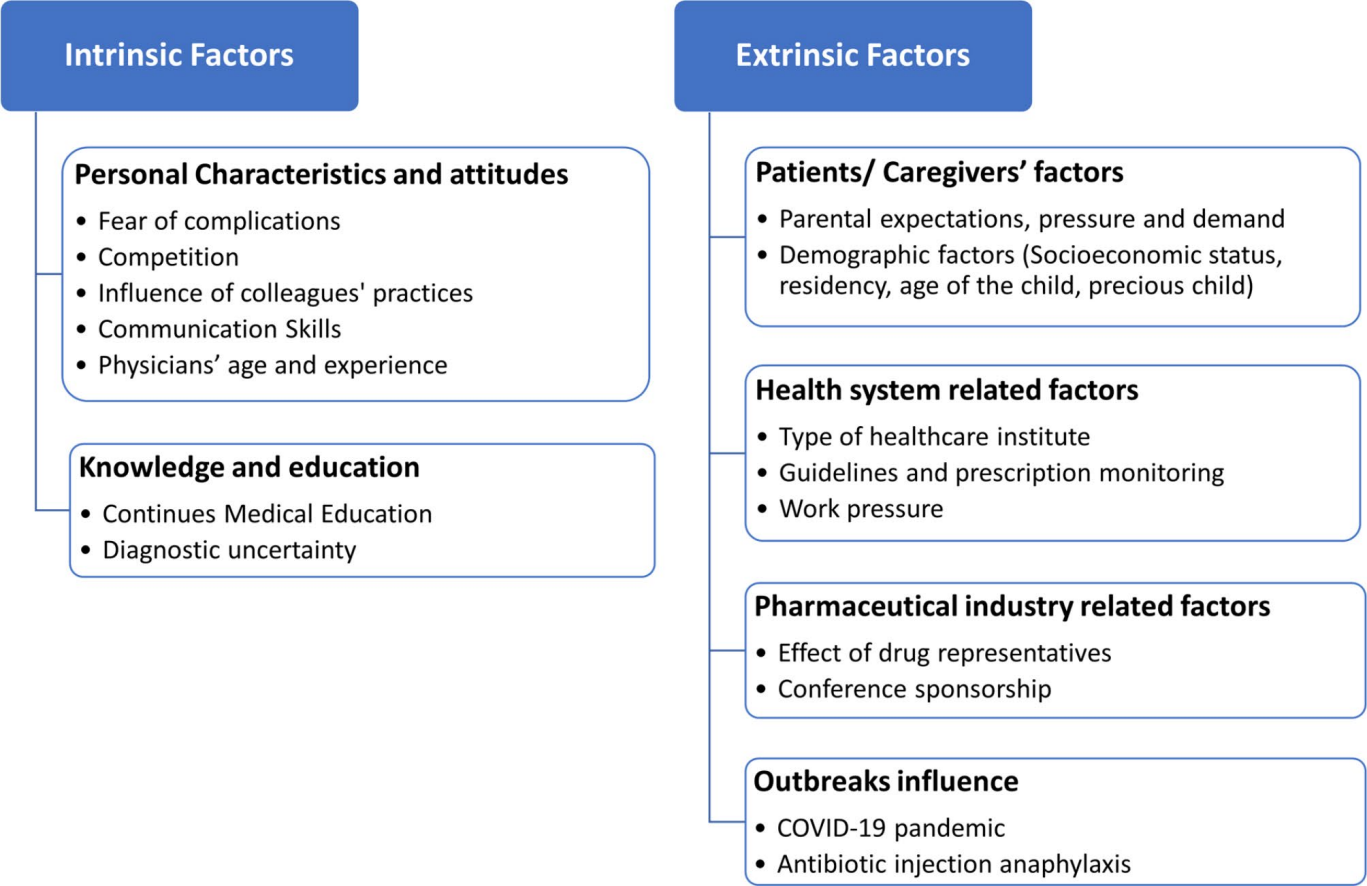
Thematic analysis of interviews on antibiotic prescribing practices: main themes and subthemes

Upon analyzing the interviews, two main themes have been mapped on our adapted theoretical framework, each comprising several subthemes. These themes align with exploring the intrinsic and extrinsic factors influencing antibiotic prescription decisions, as outlined in the interview guide. The organization of these themes and subthemes is shown in Fig. 1. Also, themes and sub-themes with their quotes were provided in Additional file 3.

### Theme 1: intrinsic factors

These factors are related to physicians and can be categorized as follows:

#### Personal characteristics and attitudes

Fear of complications has emerged as the most frequently mentioned factor influencing antibiotic prescription decisions. Participants shared that based on their own practice and observation of other physicians’ work, antibiotics were often prescribed in cases of viral infections as a precautionary measure against secondary bacterial infections. This practice was particularly prevalent in communities with low hygiene standards, or when patients and caregivers did not adhere to proper hygiene practices.

One participant explained that physicians tended to lean towards over-prescribing antibiotics rather than under-prescribing them, given the generally low levels of hygiene and nutrition in the area. The participant stated that safety was preferred, as the possibility of secondary bacterial infections was high in such circumstances.

*“Hygiene practices are severely lacking, especially in clinics located in rural areas. Due to the poor hygiene conditions in these places, doctors tend to prescribe antibiotics excessively to combat secondary bacterial infections. This fear of complications arising from the lack of hygiene is not mentioned in textbooks or guidelines, but it is highly noticeable in our context.”* (P05).

Another participant highlighted that the limited number of follow-up visits and various factors that discouraged patients from returning for re-consultation posed challenges for physicians when adopting a wait-and-see approach. Follow-up visits can be burdensome for patients and their families regarding cost, distance, and time. Consequently, physicians may find it difficult to rely solely on observation before prescribing antibiotics. Instead, they may adopt a delayed prescription approach, advising parents to start antibiotics only if the child’s condition deteriorates. This approach allowed for a balance between addressing potential bacterial infections and minimizing unnecessary antibiotic use.

*“There are instances where I only see a patient once, and I am not confident that the mother will bring the child back if complications arise or the condition persists”* (P02).

Competition was an important factor associated with physicians’ attitudes that emerged during the study. Many participants expressed concerns about this aspect. Some physicians felt compelled to prescribe antibiotics to retain patients and to consider their financial implications. They worried that if they refrained from prescribing antibiotics, patients might seek treatment from other physicians, particularly in private practice. This concern about losing patients and the potential negative impacts on their income have influenced their decision-making regarding antibiotic prescriptions.

*“However, a primary concern for physicians is to ensure patient retention and return visits. Patients pay for their clinic visits and expect to see improvement in their condition within a few days, rather than waiting for natural recovery*” (P07).

Additionally, physician characteristics include the influence of colleagues. According to an interview consultant, junior physicians tend to emulate the practices of their senior counterparts. There was a culture of learning among physicians who often observed and learned from one another. As a result, physicians’ prescription decisions can be influenced by the practices and behaviors of their peers.

*“Physicians feel compelled to prescribe antibiotics due to their widespread use among colleagues, even in cases where they may not be indicated.”* (P01).

Regarding communication skills and the physician-patient relationship, all parties agreed that effective communication is essential in addressing the issue of over-prescription of antibiotics. A physician who can effectively persuade parents that antibiotics are unnecessary, and that symptomatic treatment alone will resolve their child’s illness will not need to prescribe antibiotics to retain patients.

*“If a physician cannot effectively communicate and reassure the patient that their condition is viral and does not require antibiotics, and that further examination is available if needed, they may feel compelled to prescribe antibiotics.”* (P02).

Divergent viewpoints emerged regarding the age and years of experience of physicians. Certain individuals believe that with advancing age and accumulating experience, physicians acquire a heightened ability to distinguish between bacterial and viral infections, consequently enabling them to prescribe antibiotics with increasing precision.

*“Experience is crucial to develop the ability to differentiate between viral and bacterial infections. It enables physicians to rely on their clinical judgment and diagnostic skills to determine whether antibiotics are necessary for a patient’s specific condition.”* (P01).

In addition, junior doctors or those with less experience may prescribe more antibiotics because of the fear of losing patients.

*“Younger physicians may be more likely to prescribe antibiotics because of concerns about pediatric patients. They may feel pressure to prescribe antibiotics out of fear that parents will seek treatment from another physician.”* (P12).

However, the other participants believed that this was not a factor. They emphasized that the practice environment, good supervision, and guidance were more influential in ensuring appropriate antibiotic prescriptions.

*“Knowledge and competence are more important than age for determining a physician’s ability to differentiate between viral and bacterial infections. A young physician who has received proper education and training may be more adept at making such determinations than an older physician whose age and experience do not necessarily reflect their competency in this regard.”* (P05).

#### Knowledge and education

This sub-theme has two main points. The first is the impact of CME on prescription practices, and the second is the challenge of diagnostic uncertainty and differentiation between viral and bacterial infections.

Regarding CME, participants emphasized the importance of regularly updating their knowledge regarding antibiotic usage and distinguishing between bacterial and viral infections.

*“Continuing education is crucial to maintaining field competencies. Some individuals may have professors who do not keep themselves updated, while others who pursue advanced degrees and continue to study remain more competent.”* (P06).

Numerous participants have emphasized the importance of diagnostic uncertainty, recognizing that distinguishing between bacterial and viral infections can pose considerable challenges. In such instances, where patient follow-up may be difficult, antibiotic prescription is regarded as a precautionary approach to mitigate the risk of potentially severe complications.

*“Due to diagnostic uncertainties, many physicians struggle to differentiate between viral and bacterial infections and prescribe antibiotics as a precautionary measure. They may instruct parents to administer antibiotics if the symptoms develop. However, this approach is not suitable for all patients and is only effective for those who are educated about their condition.”* (P03).

### Theme 2: extrinsic factors

These are factors related to patients and other nonphysician factors, and can be categorized under the following subthemes:

#### Patients/caregivers’ factors

According to multiple participants, parental expectations, pressures, and demands affect physicians’ decisions to prescribe antibiotics. Certain parents believe that antibiotics yield faster outcomes, thereby prompting them to request antibiotics, particularly in the form of injections, for their children, even in situations where the underlying condition is viral.

*“Parents may express concern that their child’s condition will persist without antibiotic treatment and may request a prescription from their physician*.” (P01).

Patient demographic factors influenced physicians’ antibiotic prescription behavior. These factors encompass socioeconomic status, such as the educational background of the caregiver, as well as the cleanliness of the living conditions. When the mother or caregiver lacks knowledge or understanding, physicians may prefer to administer antibiotics directly to the patient instead of prescribing them for later use. Likewise, in unclean environments, physicians tend to lean towards providing antibiotics to patients because of the increased likelihood of bacterial or mixed infections.

*“In general, antibiotics are not prescribed when symptoms appear viral. However, the socioeconomic status of a patient may be a significant factor to consider. For example, if a child comes from an environment with poor hygiene, and the caregiver appears to be disinterested in the child’s well-being, the physician may determine that the child is at a higher risk of secondary infections. In such cases, antibiotics may be prescribed as a precautionary measure.”* (P04).

Place of residence can significantly influence antibiotic prescriptions for two primary reasons. First, proximity to medical facilities and concerns regarding the ability to monitor symptom progression can affect the decision to prescribe antibiotics. Second, the patient’s living environment, especially in rural or low socioeconomic areas, may render them more vulnerable to mixed infections.

*“Indeed, the place of residence plays a significant role in patient care. For patients residing near a medical facility, close follow-ups can be provided. However, for patients living farther away, immediate prescription of antibiotics may be necessary because physicians cannot closely monitor their condition.”* (P02).

*“In rural areas, there is a higher prevalence of mixed infections, which is why doctors often prescribe antibiotics.”* (P06).

The age of the child is also a consideration when prescribing antibiotics. For instance, children under 6 months of age are particularly susceptible to sepsis and complications, which may influence their decision to prescribe antibiotics.

*“Yes, young age, particularly in neonates, presents a high level of vulnerability as they are at risk of developing sepsis rapidly. Additionally, mothers may lack the necessary knowledge to manage such situations properly. Consequently, in cases where neonates present with a fever, antibiotics are often prescribed.”* (P12).

In certain instances, the existence of a precious child as those born after a prolonged period of infertility or a young child with a chronic illness necessitating specialized care can influence the prescription of antibiotics as a preventative measure against secondary infections. Conversely, some physicians may hesitate to prescribe antibiotics, even when warranted, due to concerns about potential parental blame in the event of complications.

*“Physicians often face pressure when treating precious infants, and this requires precautionary measures. It is widely recognized that infants are prone to complications, leading physicians to avoid treatment. This fear may result in prescription withholding or the issuance of excessive ones.”* (P05).

#### Health system-related factors

Participants raised several health system factors that impacted antibiotic prescribing practices, such as the type of healthcare institution, absence of formal national guidelines and prescription monitoring, and burden of work pressure. According to the participants, the type of healthcare institution plays a crucial role in shaping antibiotic prescription practices. In public institutions, doctors might prioritize factors other than patient satisfaction, and often rely on readily available drugs from the facility’s pharmacy when prescribing antibiotics. Conversely, physicians in private institutions may be inclined to prescribe antibiotics, even when not strictly necessary, to facilitate swift recovery and ensure high levels of patient satisfaction.

*“The approach to prescribing antibiotics differs between public and private healthcare facilities. In private practice, physicians may be more inclined to prescribe antibiotics to ensure patient satisfaction and retain patients.”* (P03).

*“In public healthcare facilities, patient satisfaction is not a primary concern, and physicians prescribe medications based on their medical appropriateness. However, in private practice, patient satisfaction becomes a significant factor, and physicians may prescribe antibiotics to ensure patient satisfaction.”* (P11).

Concerning prescription guidelines and auditing of prescriptions, participants observed the absence of formal national guidelines provided by the Ministry of Health. Consequently, prescription practices rely heavily on physicians’ individual experiences, with junior doctors often learning from their education and more experienced colleagues. Although certain healthcare institutes may have developed their own guidelines, there is typically a dearth of audit and monitoring processes to assess the appropriateness of and adherence to these guidelines.

*“There is a notable absence of official and clear guidelines, leaving individuals to seek out and gather information independently.”* (P10).

*“This presents a significant issue as there is no monitoring or control over the prescription and dispensation of medications. This problem extends beyond physicians to include pharmacies, where any medication can be dispensed and purchased by anyone.”* (P08).

However, the lack of monitoring poses a challenge. Implementing consistent adherence to clear protocols would result in a significant difference. Currently, subjective decision-making and the prescription of medications based on patient preferences are prevalent issues.” (P09).

Work pressure was identified as a factor driving physicians to prescribe antibiotics as they often face time constraints for proper disease diagnosis and explain to parents that certain conditions may be viral and do not require antibiotics.

*“Work pressure can also impact decision-making, as physicians may feel the need to complete their tasks quickly due to the high volume of patients requiring attention.”* (P11).

#### Pharmaceutical industry-related factors

Pharmaceutical companies influence antibiotic prescriptions by sponsoring conferences, offering incentives, or facilitating conference attendance for physicians.

*“Pharmaceutical companies may influence the selection of specific antibiotics or prescriptions under specific trade names. For instance, a company may offer incentives to physicians, such as conference invitations, to meet specific targets, such as prescribing a certain number of drugs. As a result, physicians may occasionally prescribe antibiotics that are not medically necessary to fulfill these targets. This situation is prevalent and occurs frequently.”* (P06).

The influence of pharmaceutical representatives on physicians’ antibiotic prescription practices varied as different participants held differing views. Depending on whether physicians rely solely on the information provided by pharmaceutical representatives without critically reviewing antibiotic details and indications, their influence can have either a positive or a negative impact on antibiotic prescriptions.

*“Pharmaceutical companies can approach physicians and promote their medications as first-line treatments for conditions, such as tonsillitis or pneumonia, claiming that their drugs are superior to others. If physicians do not critically assess this information and rely solely on it, they may be influenced by the marketing tactics employed by these companies.”* (P01)

Representatives contribute to discussions on antibiotic updates, aiming to enhance physician knowledge, while recognizing that an unbiased and accurate source of information is crucial, as more knowledge may not always be better if it is biased or flawed.

*“Another factor to consider is the role of pharmaceutical companies in providing education and training to physicians. Representatives may visit doctors to review information related to their products, serving to educate physicians and update their knowledge.”* (P03).

#### Outbreaks influence

Diverse opinions were expressed when participants were asked about the impact of the COVID-19 pandemic on antibiotic prescriptions. Some individuals believed that the pandemic had influenced antibiotic prescribing behavior, primarily because of the guidelines for managing COVID-19, which recommended azithromycin, despite it being a viral infection. Physicians perceive those antibiotics, particularly macrolides, might play a role in treating viral infections due to their potential anti-inflammatory effects.

*“Although COVID-19 is a viral infection, treatment protocols often include the use of antibiotics. This led to the perception that antibiotics play a role in treating viral respiratory infections”* (P06).

*“The COVID-19 pandemic has had a significant impact on the prescription of macrolides, which are used as anti-inflammatory agents, despite the lack of sufficient supporting evidence. Additionally, the overlap of COVID-19 with various URIs has led to the prescription of antibiotics when COVID-19 is suspected.”* (P09).

Conversely, others argued that antibiotic prescriptions remained high from the beginning and were unaffected by the pandemic.

*“The widespread use of antibiotics during COVID-19 pandemic may have exacerbated antibiotic resistance, but this is not the sole factor contributing to their frequent prescriptions.”* (P03).

*“COVID-19 pandemic has not affected the frequency of antibiotic prescriptions, as they have been prescribed frequently from the outset.”* (P12).

However, numerous participants emphasized the influence of another outbreak in addition to COVID-19, namely, frequent cases of antibiotic injection and anaphylaxis reported in Egypt over the past year, specifically related to ceftriaxone. This situation instilled fear not only among physicians but also among patients, resulting in a notable decrease in the prescription of injected antibiotics and an overall reduction in antibiotic prescriptions.

*“An outbreak of antibiotic anaphylaxis has had a significant impact on the prescription of injectable antibiotics. Physicians have become hesitant to prescribe injections, even when medically necessary, due to fear among patients. Patients may request oral medication to avoid allergic reactions, and even those who previously requested injections may refuse them. This has resulted in a noticeable change in prescribing practices.”* (P05).

*“In response to anaphylaxis, some physicians had observed improved outcomes when they stopped prescribing injectable antibiotics. This has increased awareness among physicians and led those who frequently prescribe injections to exercise increased caution and reduce their prescription rates.”* (P03).

## Discussion

The findings of this qualitative study shed light on the factors that contribute to antibiotic over-prescription in children with acute URIs as perceived by various stakeholders engaged in pediatric healthcare in Egypt. To provide a comprehensive understanding of the underlying factors, this study utilized TAPBM as a theoretical framework. The insights gained from this study can guide targeted interventions aimed at promoting rational antibiotic use and combating the escalating threat of antibiotic resistance. This study provided a comprehensive overview of antibiotic prescriptions, revealing that it is a complex process influenced by direct (physician-related) and indirect factors that impact the decision-making process.

Previous research has emphasized the significant role of physicians’ attitudes in the over-prescription of antibiotics [17, 18]. In our study, we identified two primary attitudes that contribute to this phenomenon: fear of complications and perceived competition within the medical field. The fear of complications arises from the high rate of secondary bacterial infections, as reported in studies conducted in countries with limited hygiene practices and a prevalence of mixed bacterial and viral infections, such as China and India [19, 20]. Similarly, fear of competition within the medical field, which encompasses the potential loss of patients and subsequent financial incentives, was identified as a significant concern. This issue has been observed in primary care settings in other countries, such as Korea and India [21]. In Egypt, physicians’ income is not solely reliant on their government salary but also on their private practice [22]. Thus, maintaining a steady influx of patients is crucial to sustaining their income. Consequently, when patients demand or expect antibiotics, even if they are not medically necessary, physicians may prescribe antibiotics to meet these expectations and ensure that the patients return for future visits.

One of the significant findings emphasized by the interviewed experts was the impact of the prescribing practices adopted by medical colleagues and senior professionals. The existing literature consistently acknowledges the role of peer influence in shaping the prescribing culture, wherein the clinical context and hierarchical structure within hospitals significantly influence the perceived norms and practices of various specialties, peers, and senior colleagues [23–25].

Based on the analysis in this study, effective communication skills emerged as an intrinsic factor that influenced antibiotic prescription practices. Previous studies have consistently identified communication skills as a concern, as inadequate physician-patient communication can contribute to antibiotic over-prescription [20, 26, 27]. Furthermore, research has also demonstrated that enhancing physician-patient communication can effectively reduce patient expectations for antibiotic treatment [28–30].

When considering the age and years of experience of physicians, previous research has demonstrated an inverse relationship between increased years of experience and antibiotic prescribing rates [13, 31, 32]. This finding aligns with the viewpoints expressed in the current study, as experts have reported that junior physicians often prescribe more antibiotics because of diagnostic uncertainty and fear of losing patients. However, several studies have supported the notion that age, and years of experience alone are inconclusive factors. Ongoing education and supervision of junior physicians’ practices are influential factors that can affect the prescription of antibiotics, regardless of the physician’s age or experience level [26].

Physicians’ knowledge and education are vital in shaping their antibiotic prescription practices. Previous studies have shown that CME activities and seminars, which provide training on updated antibiotic guidelines and information about antibiotic resistance, have a positive impact on physicians’ antibiotic prescription behavior [33]. CME provides physicians with new insights into the diagnosis and treatment of infectious diseases as well as updates on antibiotic regimens. The current study aligns with these findings, as many participants highlighted the positive influence of continuing medical education in helping physicians prescribe antibiotics more rationally. This has contributed to the decrease in antibiotic prescriptions.

However, diagnostic uncertainty remains a challenge, particularly when managing acute URIs in outpatient clinics. Physicians frequently encounter situations in which they must exercise clinical judgment and make decisions without a confirmed diagnosis or comprehensive understanding of the origin of the pathogen. This limitation arises because of various constraints, including time, technology, and resource constraints [34]. Although a definitive diagnosis is crucial for informing appropriate antibiotic prescriptions, it may not always be feasible to establish a diagnosis in every outpatient setting, primarily because of limitations in resources and diagnostic capabilities. Moreover, the intricacies associated with different pathogens further complicate the decision-making process for prescribing antibiotics [35, 36]. In a study conducted in China in 2019, physicians who perceived a higher occurrence of diagnostic uncertainty were more inclined to prescribe antibiotics to patients with URIs [34]. A similar pattern was observed in an Italian study, where 56% of pediatricians identified diagnostic uncertainty as the primary cause of inappropriate prescriptions [37]. The outcomes of this qualitative study align with these findings, as many participants acknowledged the difficulties associated with distinguishing between bacterial and viral infections. In uncertain situations, physicians tend to prescribe antibiotics. The over-prescription of antibiotics to children has been partly justified by the perceived risks of complications if antibiotics are not prescribed.

Extrinsic factors, including those related to patients or caregivers, significantly influence the prescription of antibiotics. Patients or caregivers can sometimes pressure physicians to prescribe antibiotics, even when they know the clinical guidelines advise against them [38]. This observation aligns with the perspectives of the participants in the current study. In pediatric patients, parental pressure and expectations have been identified as important factors contributing to inappropriate antibiotic prescriptions. A study involving 610 pediatricians found that 48% of physicians reported frequent pressure from parents to prescribe antibiotics, with 53% considering parental demand as the primary driver of over-prescription in children [39]. However, it is important to note that a study conducted in Egypt reported that a lower percentage of physicians (13.7%) cited patient pressure as the reason for antibiotic prescription [10].

Nevertheless, parental pressure and expectations significantly influence physicians’ attitudes and prescribing behaviors [13]. This observation is emphasized by certain participants in the current study, where physicians may prescribe antibiotics to fulfill parents’ expectations and ensure the patient’s continued engagement with medical care. Meeting patient expectations is a challenge. Kunin et al. (1973) referred to antibiotics as “drugs of fear,” highlighting the strong desire of physicians to utilize the latest and most potent antibiotics to address a problem and satisfy patient expectations [40].

Patient-related factors also encompass demographic variables such as residency and socioeconomic status. Within the scope of this study, several participants noted that patients from low socioeconomic backgrounds may be more susceptible to secondary bacterial infections. Moreover, limited access to healthcare facilities presents challenges in the monitoring and follow-up of patients, leading to a tendency to prescribe antibiotics as a precautionary measure against potential complications. These findings are consistent with those of a qualitative study conducted in multiple African countries, including Sudan, Guinea, and Congo. In that study, healthcare providers acknowledged consciously prescribing higher-than-recommended amounts of antibiotics due to the scarcity of follow-up visits, which were typically conducted just when patients were in critical condition. This difficulty in adopting a “wait-and-see” approach was attributed to the limited availability of follow-up care [41, 42].

Consequently, in situations of uncertainty, healthcare providers often prefer immediate antibiotic prescriptions, considering that a follow-up visit, even with assumed patient compliance, could impose a financial, logistical, or temporal burden on patients and their families. In the same study, some healthcare providers justified their preference for over-prescription over under-prescription by highlighting the generally low levels of hygiene and nutrition prevalent in the area. They believed that over-prescription would help address potential coexisting infections more effectively [41]. Furthermore, socioeconomic status may contribute to the reluctance of both healthcare providers and patients to adopt the effective “wait-and-see” or “delayed prescription” approaches commonly employed in contexts where watchful waiting and observation are feasible [42, 43].

In the context of healthcare disparities, it is essential to consider the potential impact of bias and prejudice in healthcare-related decision-making. A significant concern raised during this study is the potential over-prescription of antibiotics to children from low socioeconomic backgrounds. Although this study primarily provides qualitative insights into the factors contributing to antibiotic over-prescription, we acknowledge the pressing need for quantitative research to comprehensively assess the extent of unnecessary antibiotic prescriptions in this specific demographic. Implicit biases and prejudices can influence healthcare professionals’ clinical decisions [44]. Understanding the relationship between SES and healthcare-related decision-making is imperative to address health inequalities and ensure the equitable and rational use of antibiotics.

The healthcare system plays a pivotal role in shaping antibiotic prescription behavior, as highlighted by the participants in this study. Public healthcare facilities, where physicians are less concerned about patient retention, tend to have more rational antibiotic prescription practices than private clinics [45]. Clinical guidelines and legislation have emerged as significant factors. Healthcare systems lacking formal guidelines and policies to regulate antibiotic use, particularly in developing countries where antibiotics are available over the counter, can significantly influence antibiotic prescription behavior [18, 46]. The easy accessibility of antibiotics without a prescription can led physicians to prescribe them, even when not strictly necessary, to meet patient expectations. However, physicians may still do so responsibly, ensuring that antibiotics are administered at the appropriate dosage to minimize potential harm [8].

Moreover, the lack of prescription monitoring and feedback mechanisms within the healthcare system contributes to the unrestricted prescription of antibiotics by physicians, as they may not face legal consequences or receive feedback on their prescription practices [47, 48]. It is crucial to acknowledge that high patient volumes in healthcare institutes can also influence physicians’ decision-making regarding appropriate treatment. Rodrigues et al. addressed this aspect in their systematic review and identified a correlation between patient volume and antibiotic prescription behavior [13].

According to the interviewees, the pharmaceutical industry’s influence on antibiotic prescriptions arises from the pressure on physicians to prescribe new drugs and their financial contributions to health systems. A systematic review conducted by Rodrigues et al. examined this issue and found divergent perspectives regarding the impact of pharmaceutical company pressure. While half of the studies identified pressure as a significant factor in an antibiotic prescription, the remaining three studies reported minimal or no influence [13].

Another significant factor contributing to the prescription and overuse of specific antibiotics, particularly in the private sector, is the influence of pharmaceutical representatives. The impact of pharmaceutical companies on physicians is widely acknowledged, and legal proceedings have shed light on the extent of the industry’s influence on physicians and other stakeholders [20]. A study by Søndergaard et al. revealed that visits from pharmaceutical representatives significantly influenced doctors’ drug preferences, often favoring marketed drugs [49]. Moreover, in developing countries where ethical and legal controls may be lacking, physicians may be susceptible to the pressure exerted by pharmaceutical companies to prescribe multiple antibiotics for financial incentives [20, 50].

The findings related to the roles of the health system and the pharmaceutical industry strongly resonate with the challenges observed in the unwarranted dispensing of antibiotics within community pharmacies in Egypt. The Egyptian community pharmacy context reflects a concerning practice where subtherapeutic doses of antibiotics are readily dispensed, often under the guise of common cold products, thereby contributing to antibiotic misuse. These findings align with the broader narrative that extends beyond medical professionals and includes pharmacists who face pressures, hold beliefs about potential benefits of antibiotics, and are driven by profit motives. The ease of obtaining antibiotics from various sources and the insufficient enforcement of regulations further exacerbate the issue [51]. The behavior of community pharmacy staff, as revealed in this study, complements our understanding of the broader antibiotic misuse landscape, where patient expectations and demands can drive healthcare providers, including physicians, to prescribe antibiotics even when medically unnecessary, ultimately exacerbating the challenge of antibiotic resistance.

The impact of the COVID-19 pandemic on antibiotic prescriptions was discussed among the participants in this study. Various viewpoints have emerged regarding the influence of the pandemic on the prescribing behavior of healthcare providers with respect to antibiotics. Some participants believed that the pandemic influenced antibiotic prescriptions due to the guidelines for managing COVID-19, which recommended azithromycin despite its primary effectiveness against bacterial rather than viral infections. Two factors explain this phenomenon. First, certain studies have suggested that antibiotics, particularly macrolides such as azithromycin, may treat viral infections by exerting anti-inflammatory effects [52, 53]. Secondly, the potential prevention of complications was also mentioned, supported by other studies that reported an increased use of antibiotics during the COVID-19 pandemic due to concerns about bacterial complications or co-infections with COVID-19 [52, 54, 55]. However, other participants in our study disagreed and argued that antibiotic prescriptions remained high from the beginning and were unaffected by the pandemic. Notably, there is limited literature on the effects of the COVID-19 pandemic on antibiotic use rates, especially in children [56].

Over the past two years in Egypt, there have been numerous reported cases of antibiotic injection and anaphylaxis, particularly those associated with ceftriaxone [57]. This outbreak emerged as a significant factor in this study. The participants reported a decline in the prescription of injectable antibiotics and an overall reduction in antibiotic prescriptions. This decrease could be attributed to the fear experienced by both physicians and patients due to the reported cases.

It is important to acknowledge that qualitative research provides insights into the participants’ perceptions and experiences. However, its generalizability to a broader population may be limited. Additional quantitative studies are required to validate and quantify the impact of these factors on antibiotic prescription. Moreover, our study primarily focuses on healthcare providers, especially physicians, as key informants. While we aimed to gather insights from a diverse range of perspectives, practical challenges hindered our ability to engage with individuals from the health administrative sector, patients, and pharmaceutical companies. This limited inclusivity introduces the potential for bias in our findings, as the study predominantly represents the viewpoints of healthcare providers. We recognize that a more comprehensive exploration of various stakeholder perspectives could provide a more holistic understanding of antibiotic prescription practices.

### Recommendations

To address the multifaceted challenge of antibiotic over-prescription and to build on the findings of this study, several key recommendations can be identified. These recommendations should not only provide targeted interventions but also ensure their sustainability and active involvement of multiple stakeholders.

First, it is imperative to determine who should be involved in the implementation of these recommendations and what their specific roles should be. Healthcare professionals, including physicians and pharmacists, alongside policymakers, play central roles. Physicians, as prescribers, are integral to ensuring judicious antibiotic use. Furthermore, the involvement of thought leaders within peer groups can harness peer pressure as a force for promoting responsible antibiotic prescribing.

To make these interventions sustainable, we propose theory-based plans, which can guide and inform decision-making processes. The Theory of Planned Behavior, for instance, can provide a structured framework for evidence-based strategies aimed at changing behavior [58]. Such plans will ensure that interventions remain relevant and effective in the face of evolving healthcare dynamics.

Furthermore, the interventions should be designed in a multi-tiered fashion, targeting various aspects of antibiotic prescription practices. This approach involves a range of measures, from enhancing communication skills and continuous medical education (CME) for healthcare providers to the implementation of comprehensive national guidelines. Regulatory measures, prescription monitoring, and feedback systems are also vital components, fostering accountability and responsible antibiotic use.

To actively involve multiple stakeholders, collaboration between physicians and pharmacies should be encouraged, especially for the better use of antibiotics. This partnership ensures a unified approach in promoting rational antibiotic use and patient care.

## Conclusions

This qualitative study aimed to explore the factors contributing to antibiotic over-prescription in children with acute URIs from the perspective of experts. The analysis revealed two overarching themes: intrinsic factors related to physicians, extrinsic factors related to patients, and nonphysician influences, which together shape the prescribing behaviors.

The intrinsic factors, including physicians’ personal characteristics and attitudes, underscored the pivotal role of physician-specific attributes in driving antibiotic over-prescription. Factors such as fear of complications, limited follow-up visits, and competition within the healthcare ecosystem significantly influenced prescription practices. Moreover, the significance of physicians’ knowledge and education was evident in their decisions, while the challenge of diagnostic uncertainty further compounded the issue.

Conversely, extrinsic factors highlighted the role of patients and caregivers in driving prescription behaviors. Parental expectations and demands for antibiotics, often fueled by the belief in rapid results, stood out as influential drivers. Additionally, patients’ demographic factors, such as socioeconomic status and living conditions, played a substantial role in prescription practices. In the broader healthcare system, factors such as healthcare institution type and the absence of formal guidelines further contributed to the challenge of antibiotic over-prescription. We also noted the influence of the pharmaceutical industry and addressed the potential impact of the COVID-19 pandemic on prescription practices.

These findings bear significant implications for the field of antibiotic prescription practices. To combat antibiotic over-prescription, we recommend targeted interventions addressing both intrinsic and extrinsic factors. Incorporating study results and recommendations into practice will be instrumental in promoting responsible antibiotic use, mitigating the escalating threat of antibiotic resistance, and ultimately safeguarding public health.

### Electronic supplementary material

Below is the link to the electronic supplementary material.

Supplementary Material 1

Supplementary Material 2

Supplementary Material 3

## Data Availability

The interview transcripts generated and analyzed during the current study is not publicly available due to individual privacy possibly being compromised but is available from the corresponding author on reasonable request.

## Abbreviations

URIs: upper respiratory tract infections
AMR: antimicrobial resistance
CME: continuous medical education
IDIs: in-depth interviews
KAP: knowledge-attitude-practices
LMICs: Low- and middle-income countries
TAPBM: Teixeira antibiotic prescription behavioral model
