# Supplementary material for: Exploring factors for antibiotic over-prescription in children with acute upper respiratory tract infections in Assiut, Egypt: a qualitative study
Source: Antimicrob Resist Infect Control. 2024 Jan 7;13:2. doi: 10.1186/s13756-023-01357-2 (PMC10773027; doi:10.1186/s13756-023-01357-2)
Supplement: Supplementary file 1 — Supplementary Material 1 [file 13756_2023_1357_MOESM1_ESM.docx]

**Antibiotic Prescription in Children with Acute Upper Respiratory Tract Infections**

**Guide for Interview** *(Translated from Arabic to English):*

1. **Personal Information:**

Academic degree (latest qualification):

Job title:

Workplace:

Years of experience:

Are you working in a specific department or specialized medical unit? (Infection control, medical director, etc.)

1. **Problem Definition and Associated Consequences:**

This study aims to investigate the issue of excessive antibiotic prescription, especially in cases of upper respiratory tract infections in children.

- Do you believe this is a real problem? What are the societal consequences of this phenomenon?
- Do you personally observe this problem?

1. **Factors Contributing to Excessive Antibiotic Prescription:**

Now let's discuss the reasons why physicians tend to prescribe antibiotics without proper indication.

- In your opinion, what factors influence the medical decision-making process?
- Do the following factors affect antibiotic prescription decisions?

***Physician-related factors:***

- Age or years of experience and field of study or scientific qualifications
- Continuing medical education (conferences, workshops)
- Knowledge of appropriate antibiotic use and awareness of antimicrobial resistance
- Inability to make an accurate diagnosis
- Practices of senior colleagues
- Physician's fear of complications or lack of awareness about the consequences of antibiotic use
- Anything else?

***Patient or family-related factors:***

- Symptom severity (fever, cough, etc.)
- Patient's age, gender, birth order (precious child)
- Social status of the patient
- Patient's proximity to the healthcare facility
- Pressure or requests from the patient's family
- Physician-patient communication or communication skills
- Anything else?

***Healthcare facility or system-related factors:***

- Type of healthcare institution (public or private)
- Existence of guidelines for antibiotic prescription
- Workload pressure (patient volume and time constraints)
- Anything else?

***Pharmaceutical industry-related factors:***

- Influence of drug marketing and promotion on antibiotic prescription decisions
- Pharmaceutical companies' sponsorship of medical conferences
- Influence of pharmaceutical representatives
- Anything else?

1. **Policies and Regulations:**

- Are there any external clinic audits monitoring antibiotic prescription practices, and does their existence make a difference?
- Are there any specific policies for monitoring prescriptions, and does their existence make a difference?
- The role of infection control in addressing the issue of excessive antibiotic prescription.

1. **Impact of the COVID-19 Pandemic:**

Did the COVID-19 pandemic affect physicians' practices regarding antibiotic prescription? How?

1. **Recommendations:**

What are your suggestions for improving physicians' antibiotic prescription practices and reducing unnecessary prescriptions?

**Thank you for your participation.**
