# Supplementary material for: Exploring factors for antibiotic over-prescription in children with acute upper respiratory tract infections in Assiut, Egypt: a qualitative study
Source: Antimicrob Resist Infect Control. 2024 Jan 7;13:2. doi: 10.1186/s13756-023-01357-2 (PMC10773027; doi:10.1186/s13756-023-01357-2)
Supplement: Supplementary file 2 — Supplementary Material 2 [file 13756_2023_1357_MOESM2_ESM.pdf]

Supplementary Table 1: Themes, Subthemes, and Quotes from Thematic Analysis

<table>
<tr><th colspan="2">Problem of Over prescription</th></tr>
<tr><td colspan="2">" احنا النهاردة عندنا مشكلة كبيرة جدا ، الأطفال الي بتتحجز في العناية و الأقسام بتيجي بعدوى ما اتعودناش نشوفها و بتبقى مقاومة للأدوية الي متعودين نكتبها ما بقتش تجيب نتايج بقينا نضطر نكتب أنواع اقوى ما كناش بنستخدمها في الأطفال الا نادرا"

“Today, we are facing a very significant problem: children who are admitted to intensive care units and inpatients are developing infections that we have never seen before, and these infections have become resistant to the medications we typically prescribe. The usual drugs we used to rely on are no longer effective, so we are forced to prescribe stronger medications that we rarely used in pediatric cases.” (P05)

"بنخاف لانه ممكن يحصل resistance against organisms و برضو ليه side effects على الأطفال لأنها بتأثر على المناعة لانها حاجة بتقتل living organism و بتقتل معاه حاجات تانية زي normal flora"

“We are concerned because it is possible to develop resistance against organisms and there are also potential side effects on children from using these medications. Antibiotics affect the immune system since they kill living organisms, which can also eliminate other beneficial microorganisms such as normal flora.” (P04)</td></tr>
<tr><th colspan="2">Theme 1. Intrinsic factors</th></tr>
<tr><th colspan="2">1.1. Personal Characteristics and attitudes</th></tr>
<tr><td>– Fear of complications</td><td>"بصي هو الموضوع بيدور حوالين كذا حاجة ، أولا احنا ال hygiene عندنا قليل قوي و خصوصا في العيادات الي في القرى و المراكز او الي مستواها الاجتماعي قليل شوية بيوصفوا مضاد حيوي كتير علشان نسبة ال secondary bacterial infections كتير بسبب ان ال hygiene قليل في الأماكن دي فبيخافوا من المضاعفات دة كلام مش موجود في الكتب و لا guidelines بس ملحوظ عندنا جدا"

“Look, the issue revolves around several factors. Firstly, our hygiene practices are severely lacking, especially in clinics located in rural areas or socioeconomically disadvantaged centers. Due to poor hygiene conditions in these places, doctors tend to prescribe antibiotics excessively to combat the high incidence of secondary bacterial infections. This fear of complications arising from the lack of hygiene is not mentioned in textbooks or guidelines, but it is highly noticeable in our context.” (P05)

"مثلا ان انا العيان هشوفه مرة بس و مش ببقى واثقة قوي في الام ان هي لو حصل complication او لو طول هترجع ا وانا أصلا مش هشوف العيان تاني ، فساعات بكتبه و بقولها بصي استني يومين عالسخونة لو ما خفش ابتديه ، على أساس اخليها safe ان معاها حاجة وهي مدركة ان السخونة لو طولت عن كدة او حصل كذا او كذا تبتدأ تديه "

“there are instances where I only see a patient once and I'm not confident that the mother will bring the child back if complications arise or if the condition persists. So sometimes I write a prescription and advise her to wait for two days to see if the fever subsides before starting the medication. The intention behind this approach is to ensure the child's safety, as the mother is aware that if the fever persists or certain symptoms occur, she should administer the medication” (P02).</td></tr>
</table>

– **Competition**	"لكن المشكلة الأساسية طبعا هي انه انت عاوز تضمن ان العيان مش هيسيبك و يروح لدكتور تاني و انه هيرجع لك تاني، لان العيان بيجي العيادة بيدفع فلوس محتاج انه يطلع satisfied و يخف خلال يومين مش انه يستنى لحد ما يخف لوحده فهو بيجرب مضاد حيوي واحد و اتنين و هو أساسا بيجيلك و هو واخد حاجة من الصيدلية قبل كدة و ما نفعش"<br><br>*"However, the main problem is that the physicians want to ensure that the patient doesn't leave and go to another doctor and that they will return. The patient comes to the clinic paying money, and they need to leave satisfied and feel better within a couple of days, not wait until they get better on their own. So, the doctor tries one or two antibiotics, and the patient may have already taken something from the pharmacy before, but it didn't work." (P07)*<br><br>"و في مجال المنافسه بيجي الطفل راح عند دكتور مثلا اشتغل معاه scientific و قاله هناخد oral و فضل سخن هيسيبه و يروح لدكتور تاني طول ما الوضع تنافس هيجيلي انا و لا الدكتور اللي جنبي."<br><br>*"In the context of competition, a child may go to a doctor who, for example, worked with him scientifically and prescribed an oral medication, but the fever persisted and the child may go to another physician as long as there is competition, the child may come to me or to the doctor next to me." (P11)*
– **Influence of colleagues' practices**	"هو بيفكر مادام كله بيكتب ف انا لازم اكتب و بيشوف ناس قامات عالية و عندهم خبرة بيكتبوا antibiotics و هم عارفين أن الحالات دي غالبا حالات عادية و مش محتاجة خصة في الأطفال أما يشوفوهم هيقلدوهم "<br><br>*"He thinks that since everyone is prescribing antibiotics, he has to prescribe them too. He also sees experienced colleagues prescribe antibiotics, even though they know that these cases are not indicated, especially in children. When he sees them doing it, he imitates them." (P01)*<br><br>"في كذا سبب رقم واحد اتعلم من حد تاني غلط عشان شاف حد كبير بيعمل كده"<br><br>*"…there are several reasons why people learn wrong practices from others, but one significant reason is when they observe someone senior or experienced engaging in those practices." (P13)*
– **Communication Skills**	"طبعا لو الدكتور مش هيعرف يعمل reassurance كويس و يفهم العيان اند viral ة و مش محتاج مضاد حيوي و ان انا متاح لو احتجت تكشف تاني، لو انت مش هتعرف توصل الكلام دة بطريقة تخلي النريض assured يبقى مش هتعرف الا انك تكتبله مضاد حيوي"<br><br>*"Of course, if the doctor is unable to provide good reassurance and make the patient understand that their condition is viral and does not require antibiotics, and that I am available for further examination if needed, if you are unable to convey this message in a way that reassures the patient, then you will have no choice but to prescribe antibiotics." (P02)*

	"في العيادات ٩٩.٩ communication و .١ طب، مدى نجاحه و رضا العيان بيعتمد على الcommunication مع العيان مش الطب."<br><br>*“In clinics, 99.9% of success relies on communication, while only 0.1% relies on medical expertise. The patient's satisfaction and trust are primarily based on the communication with the healthcare provider rather than solely on medical knowledge.” (P03)*<br><br>" لانه احيانا بتيجي الأمهات بتبقى عارفه أن ابنها هيتكتبله antibiotics ف لو حد مش عنده communication هيفهمها أن دا مش صح مش هيكتب و خلاص و العكس أن في امهات بتبقى مش عايزه antibiotics و الحالة محتاجه ف لو communication كويس هيكتبله."<br><br>*"Sometimes, mothers come to the clinic with the preconception that their child will be prescribed antibiotics. If a healthcare provider lacks effective communication skills, they may fail to convince the mother that antibiotics are unnecessary. On the other hand, some mothers may not want antibiotics even when the child needs them, and good communication skills can help persuade them otherwise." (P06)*
– **Physicians’ age and experience**	"اكيد، الخبرة دورها كبير في القدرة على التفريق الفيرس و البكتيريا. الخبرة بتسمح للدكاترة إنهم يعتمدوا على الكشف و خبرتهم في التشخيص عشان يعرفوا إزاي يحددوا إذا كان في حاجة لمضاد حيوي أو لا"<br><br>*“Exactly, experience plays a crucial role in developing the ability to differentiate between viral and bacterial infections. Experience enables them to rely on clinical judgment and diagnostic skills to determine whether or not antibiotics are necessary for a patient's specific condition.” (P01)*<br><br>"فرق اكيد الصغيرين بيوصفوا اكتر و دا عشان خوفهم على العيانين لانه بيقول هيسيبوني و يروحوا لدكتور فلان الي هيكتب مضاد حيوي"<br><br>*“Yes, it is true that younger physicians tend to prescribe antibiotics more frequently, and this is often due to their concern for their young patients. They may feel pressure to prescribe antibiotics because they fear that parents will take their children to another doctor who might prescribe them if they do not.” (P12)*<br><br>"هو السن مش هيفرق قد ما حد علمه صح ولا لا، نائب صغير حظه حلو في مكان حلو حد هيعلمه (خد بالك دي فيرال دي بكيتريا دي هديها antibiotics كذا) ممكن تلاقي ناس سنها كبير و خبرتها على قدها"<br><br>*“Age doesn't make a difference as much as someone's knowledge and competence. A young doctor could be fortunate to receive proper education and training in a good environment where someone will teach them (be careful, this is viral, this is bacterial, prescribe antibiotics in this case). On the other hand, you may find older individuals whose age and experience do not necessarily reflect their competency in this regard.” (P05)*

<table>
<tr><th colspan="2">1.2. Knowledge and education</th></tr>
<tr><td>– Continues Medical Education</td><td>
"اكيد طبعا قصة اني اخد شهادة و ابني عليها موضوع سيء و لازم يجدد المعرفة و الupdates و يراجع على المعلومات بتاعته يمكن احنا على أساس أن احنا مشغلين مدربين بنعملهم refresh و updated و لكن بعض الزملاء بياخدوا الشهادة و يناموا عليها لأنهم فتحوا العيادة و شغالين في المستشفى"

"Of course, the story of getting a degree and relying on it is a bad practice. It is necessary to renew knowledge, stay updated, and review the information regularly. Our engagement in training and education keep us updated, but some colleagues may acquire a degree and then neglecting the need for ongoing learning and relying solely on their initial qualification while working in clinics or hospitals." (P04)

"شوفت ناس أساتذته في المجال و مش update و في ناس خدت ماستر بس قاري بيبقى احسن "

"I've seen people whose professors in the field did not keep themselves updated, while others who pursued a master's degree and continued to study remained more competent." (P06)
</td></tr>
<tr><td>– Diagnostic uncertainty</td><td>
"معظمنا مش بيكون عارف ، احنا حتى لما بنقول ان دة viral بنقول للناس ارجعوا بعد يومين تلاتة، معظمنا مش بيكون متاكد لان مش بنعمل أي تحاليل تقولنا حتى دة viral و لا لا "

"Most of us are not sure. Even when we tell people that it's viral, we usually advise them to come back after two or three days. We are not confident because we don't conduct any tests to confirm whether it's viral or not." (P06)

"التفريق بين العدوى الفيروسية او البكتيرية صعب و في overlapping بينهم و مش اي حد يعرف يفرق بينهم كل الكتب تقول اعمل culture أو gram stain بس ده مش موجود على أرض الواقع ف مفيش وسيلة تخليك تفرق بسرعة من غير ما نستنى ال culture الي بتاخد وقت طويل"

"Differentiating between viral and bacterial infections is challenging due to overlapping symptoms, and there is a lack of rapid diagnostic tools available to quickly distinguish between them without relying on time-consuming culture tests." (P08)

"ممكن مثلا طفل يشتكي من حمى و throat sore بيضطر الدكتور يدي مضاد حيوي و يظهر بعد ٥ ايام rash دا معروف فيرس و الدكاتره بيستغل جهل العيان و يقولوا طالما طفح يبقى خف"
"a child complaining of fever and sore throat even may receive antibiotics from the doctor, and after five days, a rash appears, indicating a viral infection. Some doctors take advantage of the parent's lack of knowledge and claim that it was a bacterial infection and the rash is a sign of improvement." (P11)

"دكاترة كتير بتلاقي صعوبة في التفريق بينهم فبيكتبوا المضاد في الروشته احتياطي و بقيول للام لو كذا ظهر ادي المضاد الحيوي بس دة مش هينفع مع كل العيانين مع الeducated بس"
"many doctors struggle to differentiate between viral and bacterial infections, leading to some prescribing antibiotics as a precautionary measure due to diagnostic uncertainties. They may include antibiotics in the prescription, instructing parents to administer them if certain symptoms develop. However, this approach is not suitable for all patients, only those who are educated." (P03)
</td></tr>
</table>

Theme 2. Extrinsic factors

2.1. Patients/ Caregivers’ factors

– **Parental expectations, pressure and demand**	"الأهالي بيقلقوا إن لو ما أخدش طفلهم مضاد حيوي، هيبقى حالته مستمرة و هيستمروا في المعاناة. الخوف ده بيخليهم يطلبوا مضاد حيوي. في بعض الحالات، حتى لو الطفل اخد علاج كذا يوم بدون تحسن، الاهل بيروحوا لدكتور تاني يكتبلهم مضاد حيوي"<br><br>*“Parents express concerns that if their child does not receive antibiotics, their condition will persist, and they will continue to suffer. This fear drives them to request antibiotic prescriptions from doctors. In some cases, even if the child has already undergone treatment for a few days without improvement, the parents may seek another doctor who is more likely to prescribe antibiotics.” (P01)*<br><br>"هو بيجيلك تاني واحد بعد ما جرب قبل كدة فانت علشان تضمن انه يجيلك تاني لازم ترضيه فانت بتكتب الحاجة الجديدة او الي بتكون اقوى و برده للأسف مفهوم الناس ان الي بينجز و يخفف اسرع هو الحقن و الفكرة دي مغلوطة طبعا ، بس الدكاترة بتكتب حقن حتى لو مش مطلوبة بس علشان مفهوم الناس"<br><br>*“The patient comes expecting to receive a new medication, so you need to satisfy their expectations. You write the new prescription or something stronger. Unfortunately, people have a misconception that antibiotics administered by injection provide faster relief. Of course, this idea is incorrect, but doctors still prescribe injections even if they are not necessary, simply to meet people's expectations” (P05)*
– **Demographic factors (Socioeconomic status, residency, age of the child, precious child)**	"حسب الاعراض ال symptoms لان لو باين انه viral مش هنكتب بس ممكن حسب ال socioeconomic بتاع العيان يعني دة عامل مهم، لما بشوف الطفل دة جاي و ال hygiene وحش قوي ا وان الام باين انها ignorant و مش هتعرف تتعامل مع الطفل او مش مهتمة بيه و انا عارف البيئة الي جاي منها مش نضيفة فانا عامل حسابي ان دة هيتعرض ل secondary infection بسهولة بايدي بتبقى اخف شوية اني اكتب مضاد حيوي"<br><br>*“In general, when the symptoms appear to be viral, antibiotics are not prescribed. However, depending on the socioeconomic status of the patient, it can be a significant factor to consider. For instance, if it is observed that the child is coming from a place with very poor hygiene and the mother seems ignorant or disinterested in taking care of the child, and the physician aware that the environment they come from is not clean, then it is considered the child may be more prone to secondary infections. In such cases, antibiotic may be prescribed as a precautionary measure.” (P04)*<br><br>" لما كمان بيكونو low socioeconomic مش هيقدروا يكشفوا برة و لا يشتروا المضاد الحيوي لما يبقو محتاجينه فيصرفوه من الصيدلية هنا و ياخده قبل ما يروح بحيث يخليه معاه و ياخده لما يحتاجه حتى لو حد بعد كدة وصفه لهم"<br><br>*“for low socioeconomic individuals, they may not be able to afford medical examinations or purchase antibiotics when they need them. They end up getting them dispensed outpatient pharmacy, keeping them for future use, and taking them when needed, even if it's prescribed by another doctor later on.” (P08)*

Demographic factors (Socioeconomic status, residency, age of the child, precious child)	" يعني مثلا tonsillitis follicular تبدأ congestion و low grade fever ممكن تكمل كده و ممكن تقلب high grade / pus ف بقولها بنكمل على symptomatic treatment مضاد أو حاجة للسخونية و لو عدت الحرارة ٣٨.٥ ابتدي ادي ده ف في ام فاهمه هتتابع و هتشوف ، و في ام مش فاهمه ف هنديها العلاج من الاول كده." <br>*"For example, in the case of follicular tonsillitis, it starts with congestion and low-grade fever, which can either continue like that or progress to pus formation and high-grade fever. In such cases, I would recommend symptomatic treatment, such as antipyretics. If the temperature exceeds 38.5 C^{o}, I would start giving them antibiotics. If the mother understands and follows up, she will observe the progress. However, if the mother doesn't understand, I would provide the antibiotic from the beginning"* (P03) <br>"طبعا مكان السكن بيفرق لأن القريب نعمله close follow up هقوله تعالى بكره او بعد يومين ف هعرف لو دخل في secondary bacterial infections و على فكره هي كتيرة جدا و احنا ك أطباء بيجبلنا rhinorrhea و يقلب ب sore throat ف البعيد ممكن اديله لاني مش هعرف اعمل close observation و ممكن أقوله لو حصل كذا و كذا خد المضاد بس في ناس بتريح دماغها و تكتبه من الأول" *"Indeed, the place of residence does make a difference because for patients who live nearby, we can provide close follow-up. For patients who live far away, antibiotics may be prescribed immediately because physician will not be able to closely observe their condition. some physicians may use delayed prescriptions and informed parents that if certain symptoms occur, they should take the prescribed antibiotics. Others find it reassuring to have the prescription from the beginning."* (P02) <br>طبعا لان القرى فيها mixed infection كتير دا بيؤدي أننا نكتب antibiotics *"In rural areas, there is a higher prevalence of mixed infections, which is why doctors often prescribe antibiotics."* (P06) <br>"مع السن الصغير قوي ال neonates بالذات محدش بيامن لهم يعني بيقلبوا sepsis بسرعة جدا و الأمهات مش بتعرف تتعامل مع الحالة كويس ، فال neonate ايوة الناس بتدي مادام سخن يوم بيكتب مضاد حيوي ففي وصف antibiotics كتير ليهم" <br>*"Yes, young age, especially neonates, are very vulnerable as they can quickly develop sepsis. Moreover, mothers often lack proper knowledge on how to handle such situations. So, when it comes to neonates, if they have a fever, an antibiotic is prescribed."* (P12) <br>"الدكاترة في الprecious baby دايما في ضغط ف لازم ناخد احتياطاتنا و الام بتكون جاية راحت لدكاترة كتير و سألت كتير و خدت كذا علاج و معروف دائما أن الprecious baby دايما complication و بعض الأطباء بيقولوا precious ابعدني عنه و الخوف شديد يؤدي لعدم كتابة أو يؤدي لكتابة بزيادة" *"The doctors are always under pressure when it comes to precious babies, so we need to take precautions. The mother has been to many doctors, asked many questions, and tried several treatments. It is well-known that precious babies always have complications, and some doctors preferred to not treating them. The fear leading to either not writing prescriptions or writing excessive ones."* (P05)

2.2. Health system related factors	
– **Type of healthcare institute**	"هو جوة المستشفات الحكومي احنا محكومين بكام نوع حسب الموجود في الصيدلية و في الغالب بتبقى حاجات بسيطة قوي دول الي الدكاترة ملتزمين بيهم ، اما في البرايفت فالناس ايديها سايبة الصراحة و بتكتب أنواع جديدة و اقوى حتى لو مش indicated"<br><br>*"In public hospitals, our options for prescribing are limited to the available medications in the pharmacy. Usually, these options are quite limited, and physicians tend to stick to a few commonly used ones. However, in private practice, physicians have more freedom to choose medications and frequently prescribe new generations of antibiotics even if not indicated"* (P05)<br><br>"بتفرق في الخاص بيكتب اكتر علشان ما يخسرش العيان"<br><br>*"it differs, in private practice, physicians may tend to prescribe antibiotics, to ensure patient satisfaction and avoid losing patients."* (P03)<br><br>"الحكومي مش هيكتب مش فارق معاه رضا العيان اوي ف مش هيكتب لانه عارف أن دا الصح ، لكن في الخاص اهم حاجة رضا العيان فهيكتبله عشان ميخسرش العيان."<br><br>*"In public healthcare facilities patient satisfaction is not a concern, so physicians do not prescribe based on patient satisfaction. They prescribe based on what they know is medically appropriate. However, in private practice, patient satisfaction becomes a significant factor, and physicians may prescribe antibiotics, to ensure patient satisfaction and retain patients."* (P11)
– **Guidelines and prescription monitoring**	"لا مفيش حاجة رسمية و واضحة ، الي عاوز يدور هيلاقي"<br><br>*"There are no official and clear guidelines available. Those who seek it will have to search and find information on their own."* (P10)<br><br>"و دي اكبر مشكلة مفيش أي رقابة على الي بيتكتب و بيتوصف و المشكلة مش الدكاترة بس المشكلة في الصيدليات كمان ان أي حاجة ممكن تتصرف و أي حد يقدر يشتريها "<br>*"This is a significant issue as there is no monitoring or control over prescriptions and dispensing. The problem extends not only to physicians but also to pharmacies. Anything can be dispensed, and anyone can purchase it"* (P08)<br><br>"مفيش مراقبة للاسف و اكيد لو في بروتوكول كله يمشي عليه هيفرق طبعا بدل أن كله ماشي بمزاجه و اللي عايزه المريض فبيعمل فروق شخصيه"<br>*"Unfortunately, there is a lack of monitoring. If there were clear protocols in place and followed consistently, it would certainly make a difference. Currently, there is a problem of subjective decision-making and doctors prescribing based on patient preferences."* (P09)

– **Work pressure**	"عدد العيانين كتير و في نفس الوقت الدكتور بيقول لو كل حالة نعملها تحاليل مش هنخلص"<br>*"The number of cases is high, and at the same time, physicians often mention that if they were to conduct tests for every case to determine if an antibiotic is necessary or not, it would be time-consuming and impractical." (P02)*<br>"ضغط الشغل بيفرق لان الدكاترة بيحسوا انهم عاوزين يخلصوا شغلهم بسرعة علشان يلاحقوا على كل العيانين"<br>*"Work pressure can have an impact, as physicians may sometimes feel the need to complete their tasks quickly due to the high number of patients they need to attend to." (P11)*<br>"اما يلاقي زحمة هيدي عشان يخلص او ممكن ميديش خالص لمريض محتاج ف قراراته مش هتبقى مظبوطة"<br>*"When faced with a heavy workload, physicians may prescribe antibiotics to finish the process and move on to the next patient. Alternatively, in some cases, they may not prescribe antibiotics when they are necessary, leading to inaccurate decision-making due to the workload pressures." (P07)*
**2.3. Pharmaceutical industry related factors**	
– **Effect of drug representatives**<br>– **Conference sponsorship**	"بتفرق اكتر في اختيار نوع المضاد الحيوي او الشركة الي هو ال trade name بس طبعا بياثر على كتابة الروشتات يعني ممكن الشركة علشان تطلعني مؤتمر لازم أوصل لتارجت معين فاوقات هكتب لعيانين مش محتاجين علشان أوصل للتارجت دة، فدي موجودة و بتحصل"<br>*" pharmaceutical companies may affect the selection of a specific antibiotic or a prescribing a specific trade name. For example, a company might offer physicians conference invitations if they meet a specific target, such as prescribing a certain number of their drugs. As a result, physicians may occasionally prescribe antibiotics that patients do not actually require in order to fulfill this target. This situation is prevalent and occurs frequently." (P06)*<br>"ممكن يدخل للدكتور يعمل من البحر طحينة احنا first line treatment لل tonsillitis أو pneumonia أو غيرها و الدوا بتاعي دا سحر و بيظبط كله و احسن من كذا و كذا ف لو الدكتور مقراش و خد كلامه هيبقى عامل زي الكورة اللي في ايد كل شركة و بيدخلوا على البرايفت اكتر بيطلبوا منه يطلع كذا علبه و ليك نسبة ف ليهم تأثير في حوار الدوا"<br>*"It is possible for a representative to approach a physician and told them that their medication is the first-line treatment for conditions such as tonsillitis or pneumonia, claiming that their drug is magical and superior to others. If the doctor does not critically assess the information and solely relies on it, they may end up being influenced by the marketing tactics employed by various pharmaceutical companies" (P01)*<br>"يعني بيقوله ايه المادة الفعاله و بيراجع مع الindications و مش هتاثر على قرار الدكتور و في نقطة كمان أن في شركات أدوية بتعلم الدكتور و خاصه الجونيور بحيث أن المندوب بييجي يراجع مع الدكتور كل المعلومات المتعلقة بالمنتج ف دا بيعمل awareness للدكتور و يعمل update لمعلوماته"<br>*"Another point to consider is that pharmaceutical companies also provide education and training to doctors, particularly junior physicians. The representative visits the doctor to review all the information related to the product. This process helps to educate the physicians and update their knowledge" (P03)*

**2.4. Outbreaks influence**	
**COVID-19 pandemic**	"دة حقيقي و COVID 19 رغم أنه viral و لكن protocol فيه antibiotics و مختارين اقوى الأنواع ف خلى الناس قلبها تقيل مهو viral و بكتبله antibiotics اهو ، لكن COVID كان بيبقى complicated ب bacteria و بيعمل تأثير كبير على الرئة و احنا بنحاف على العيان من اي complications"<br>*"That's true, and despite COVID-19 being a viral infection, the protocol includes antibiotics. This has led people to believe that antibiotics has a role in respiratory viral infections. However, COVID-19 can become complicated with bacterial infections and have a significant impact on the lungs, which is why we are cautious about any potential complications." (P06)*<br><br>"الكورونا خلى في اعتقاد عند الدكاتره أن الazithromycin ليها طريقة معينة بتوقف الviral مش لازم يبقى mixed أو bacterial و دي هزت القناعة بتاعت أن الفيرال مش بياخد مضادات حيوية"<br>*"COVID-19 has led physicians to believe that Azithromycin has a specific way of stopping viral infections without the need for a mixture or bacterial involvement. This has shaken the conviction that viral infections do not require antibiotics." (P03)*<br><br>"أثرت جدا و خصوصا في كتابة الماكروليدز لأننا كنا بنستخدمها ك antiinflammatory و متجرب من غير دراسة ف دعم الفكرة دي و في نفس الوقت الكورونا متداخله مع كل الupper ف كان بيتكتب اول ما نشك في كورونا فدة أثر جدا "<br>*"It had a significant impact, especially in the prescription of macrolides, as it used as anti-inflammatory agents without sufficient studies supporting this concept. Additionally, COVID-19 overlapped with various upper respiratory tract infections, so when COVID-19 is suspected, they prescribe antibiotics." (P09)*<br><br>"اعتقدش كدة كدة بيتكتب كتير هو الي حصل فترة كرونا زود المشكلة اكتر لان كل الناس بقت تاخد antibiotics فدة زود مشكلة ال resistance بس مش هو السبب كدة كدة بيتكتب كتير"<br>*"I don't believe it has had an impact on antibiotic prescriptions. The situation during the COVID-19 period worsened the issue of antibiotic resistance as a result of widespread antibiotic use. However, this is not the sole factor contributing to its frequent prescription" (P03)*<br><br>"لا مفرقتش لاننا من البداية بنعمل كده"<br>*"No, it didn't make a difference because antibiotics have been prescribed frequently from the beginning." (P12)*

- **Antibiotic injection anaphylaxis**	"اعتقد ايوة ، بس الي انا شفته اثر اكتر حوار ال anaphylaxis الي حصل، الدكاترة بقوا بيخافو يكتبو injections حتى الناس نفسها حتى لو مطلوب injection هم بيخافوا و بيقولنا لا لو ممكن نكتب دوا شرب علشان الحساسية، هم الناس الي كانووب يطلبوا الحقن دلوقتي بقوا بيرفضوها ،ففرق فعلا"<br>*"What I observed had a greater impact is antibiotic anaphylaxis outbreak. Physicians became afraid to prescribe injections, even when they were necessary, because even the patients themselves were scared. They would tell us, "No, if possible, prescribe oral medication to avoid any allergic reactions." Now people who used to request injections are refusing them. There has definitely been a noticeable change." (P05)*<br><br>"و نقطة الanaphylactic الدكاتره لقوا لما بطلوا يدوا injection عمال على بطال و النتائج بتتحسن ف دا زود الوعي عندهم ف اللي عنده habit أنه بيكتب كتير يخاف ميكتبش بس لما شاف الموضوع ده بقى يقلل"<br>*"Regarding the point about anaphylaxis, physicians found that when they stopped giving injections, there was a noticeable improvement in results. This increased their awareness, and those who had a habit of prescribing frequently became cautious and reduced their prescription rate." (P03)*
