# Supplementary material for: Exploring factors for antibiotic over-prescription in children with acute upper respiratory tract infections in Assiut, Egypt: a qualitative study
Source: Antimicrob Resist Infect Control. 2024 Jan 7;13:2. doi: 10.1186/s13756-023-01357-2 (PMC10773027; doi:10.1186/s13756-023-01357-2)
Supplement: Supplementary file 3 — Supplementary Material 3 [file 13756_2023_1357_MOESM3_ESM.docx]

**Additional file 1. COREQ (Consolidated criteria for reporting qualitative studies): 32‐item checklist.**

| **Number & Item** | **Guide questions and description** | **Additional comments** | **Reported on page no. or**  **not applicable (N/A)** |
| --- | --- | --- | --- |
| **Domain 1: Research team and reﬂexivity** | | | |
| *Personal Characteristics* | | | |
| 1. Interviewer/  facilitator | Which author/s conducted the interview or focus group? | MA | Methods, pg. 7 |
| 2. Credentials | What were the researcher’s credentials? E.g. PhD, MD | PhD candidate | N/A |
| 3. Occupation | What was their occupation at the time of the study? | Assistant lecturer of public health | N/A |
| 4. Gender | Was the researcher male or female? | Female | N/A |
| 5. Experience and training | What experience or training did the researcher have? | MA had a previous experience in qualitative research as she is conduct IDIs and FGDs for previous qualitative research. She also attend online courses to learn thematic analysis. | N/A |
| *Relationship with participants* | | | |
| 6. Relationship established | Was a relationship established prior to study commencement? | No. | N/A |
| 7. Participant knowledge of the interviewer | What did the participants know about the researcher? e.g. personal goals, reasons for doing the research | MA was not known to the participants, her role and function, as well as the goal and procedure of the interviews, were explained to the participants in the informed consent beforehand and declared again in the opening remarks before the interview began. | N/A |
| 8. Interviewer characteristics | What characteristics were reported about the interviewer/facilitator? e.g. Bias, assumptions, reasons and interests in the research topic | MA, the interviewer, is a doctoral candidate with a medical background as a physician.  This combination of roles and expertise may increase awareness of specific biases and assumptions fostering reflexivity in the study | Methods, pg. 8 |
| **Domain 2: study design** | | | |
| *Theoretical framework* | | | |
| 9. Methodological orientation and Theory | What methodological orientation was stated to underpin the study? e.g. grounded theory, discourse analysis, ethnography, phenomenology, content analysis | Data were analyzed using thematic analysis according to Braun and Clarke guidlines | Methods, pg.8 |
| *Participant selection* | | | |
| 10. Sampling | How were participants selected? e.g. purposive, convenience, consecutive, snowball | Purposive sampling | Methods, pg. 6 |
| 11. Method of approach | How were participants approached? e.g. face-to-face, telephone, mail, email | Face to face | Methods, pg. 7 |
| 12. Sample size | How many participants were in the study? | 13 participants. | Methods, pg.7 |
| 13. Non-participation | How many people refused to participate or dropped out? Reasons? | No one dropped out. | N/A |
| *Setting* | | | |
| 14. Setting of data collection | Where was the data collected? e.g. home, clinic, workplace | The interviews were conducted in participants’ workplaces | Methods, pg.7 |
| 15. Presence of non-participants | Was anyone else present besides the participants and researchers? | Besides the researcher and participant, no one else was present during the interviews. | N/A |
| 16. Description of sample | What are the important characteristics of the sample? e.g. demographic data, date | Fully presented in Methods section (Table 1) | Methods, p. 6 |
| *Data collection* | | | |
| 17. Interview guide | Were questions, prompts, guides provided by the authors?  Was it pilot tested? | The interview guide is provided as supplementary material (translated from Arabic to English). | Supplementary file 2 |
| 18. Repeat interviews | Were repeat interviews carried out? If yes, how many? | No repeat interviews were carried out. | N/A |
| 19. Audio/visual recording | Did the research use audio or visual recording to collect the data? | The interviews were audio-recorded. | Methods, pg.7 |
| 20. Field notes | Were ﬁeld notes made during and/or after the inter view or focus group? | No | N/A |
| 21. Duration | What was the duration of the interviews or focus group? | On average, one interview lasted 20-40 minutes | Methods, pg. 7 |
| 22. Data saturation | Was data saturation discussed? | Yes | Methods, pg. 7 |
| 23. Transcripts returned | Were transcripts returned to participants for comment and/or correction? | The transcripts were not returned. | N/A |
| **Domain 3: analysis and ﬁndings** | | | |
| *Data analysis* | | | |
| 24. Number of data coders | How many data coders coded the data? | The interviewer MA coded the data and coded revised by AFE and this discussed their findings with other researchers in the research team. | Methods, pg.8 |
| 25. Description of the coding tree | Did authors provide a description of the coding tree? | No coding tree is provided. | N/A |
| 26. Derivation of themes | Were themes identiﬁed in advance or derived from the data? | Inductive thematic analysis was conducted, wherein similar codes were combined to create major categories using a thematic map. This process led to identifying new themes .These themes were then mapped deductively against TAPB model. | Methods, pg. 8 |
| 27. Software | What software, if applicable, was used to manage the data? | MAXQDA 202 | Methods, pg. 8 |
| 28. Participant checking | Did participants provide feedback on the ﬁndings? | Yes, a member check was carried out for five participants who were randomly selected from the list of participants. They were asked to confirm that the findings of the analysis and the interpretations were consistent with their experiences and perspectives. | Methods, pg.8 |
| *Reporting* | | | |
| 29. Quotations presented | Were participant quotations presented to illustrate the themes/ﬁndings? Was each quotation identiﬁed? e.g. participant number | Yes, participant quotations are presented to illustrate the themes and each quotation is identified by participant number. | Resultss, pg. 9 |
| 30. Data and ﬁndings consistent | Was there consistency between the data presented and the ﬁndings? | The results are based on the interview statements | Resultss, pg. 9 |
| 31. Clarity of major themes | Were major themes clearly presented in the ﬁndings? | Yes, major themes are clearly identified. | Resultss, pg. 9 and Figure 1. |
| 32. Clarity of minor themes | Is there a description of diverse cases or discussion of minor themes? | Yes, minor themes are clearly identified. | Resultss, pg. 9 and Figure 1. |
